# Review of Predicting Synergistic Drug Combinations

**DOI:** 10.3390/life13091878

**Published:** 2023-09-07

**Authors:** Yichen Pan, Haotian Ren, Liang Lan, Yixue Li, Tao Huang

**Affiliations:** 1 Bio-Med Big Data Center, CAS Key Laboratory of Computational Biology, Shanghai Institute of Nutrition and Health, University of Chinese Academy of Sciences, Chinese Academy of Sciences, Shanghai 200031, China; panyichen2023@sinh.ac.cn (Y.P.); renhaotian2021@sibs.ac.cn (H.R.); 2 Department of Interactive Media, Hong Kong Baptist University, Hong Kong, China; lanliang@hkbu.edu.hk; 3 Key Laboratory of Systems Health Science of Zhejiang Province, School of Life Science, Hangzhou Institute for Advanced Study, University of Chinese Academy of Sciences, Hangzhou 310024, China; 4 Guangzhou Laboratory, Guangzhou 510005, China; 5 School of Life Sciences and Biotechnology, Shanghai Jiao Tong University, Shanghai 200240, China; 6 Collaborative Innovation Center for Genetics and Development, Fudan University, Shanghai 200433, China

**Keywords:** drug combination, synergistic effect, drug resistance, side effects, machine learning, deep learning

## Abstract

The prediction of drug combinations is of great clinical significance. In many diseases, such as high blood pressure, diabetes, and stomach ulcers, the simultaneous use of two or more drugs has shown clear efficacy. It has greatly reduced the progression of drug resistance. This review presents the latest applications of methods for predicting the effects of drug combinations and the bioactivity databases commonly used in drug combination prediction. These studies have played a significant role in developing precision therapy. We first describe the concept of synergy. we study various publicly available databases for drug combination prediction tasks. Next, we introduce five algorithms applied to drug combinatorial prediction, which include traditional machine learning methods, deep learning methods, mathematical methods, systems biology methods and search algorithms. In the end, we sum up the difficulties encountered in prediction models.

## 1. Introduction

As a new cross-discipline, pharmacogenomics mainly studies how genomic changes affect drug response and explains the role of drugs in clinical treatment. So far, it is still challenging to use transcriptome data to predict drug responses in tumors because of the heterogeneity between cell lines and tumor cells [1]. Cell lines are cells grown in the laboratory from tumor tissue, while tumor cells are cells in real tumor tissue. Genomic and epigenetic differences exist between cell lines and tumor cells, including mutation, copy number variation, DNA methylation, histone modification, etc. Therefore, there are some differences in cell state, metabolic characteristics and cell signaling pathways between cell lines and tumor cells. This heterogeneity makes it challenging to use transcriptome data from cell lines to predict drug response in tumors. In predicting the sensitivity of melanoma to drugs, Barretina J. et al. [2] found that when applied exclusively to melanoma-derived cell lines, classifiers built using entire cell line datasets performed poorly, with a true positive of only about 0.6 when the false positive was 0.2. Models built using only melanoma cell lines performed better on the receiver operating characteristic (ROC) curve, with a true positive of 0.8 when the false positive was 0.2.

Similarly, there is considerable heterogeneity in inter-tumor and intra-tumor cells. Even patients with the same cancer type may have different prognoses under the same clinical treatment. Tumor heterogeneity makes tumor drug resistance become an urgent problem to be solved. Drug resistance is mainly caused by these mechanisms, such as the mutation of drug target [3], increased efflux of drugs [4] and amplification of an alternate pathway [5]. The main strategy to overcome tumor drug resistance is combination therapy. Combination therapy uses a variety of drugs in the process of treatment, which not only reduces drug intake and side effects but also improves the therapeutic effect by targeting multiple genes and pathways at the same time [6].

In vivo, animal experiments and in vitro drug screening for “case-by-case” identification are the main source of traditional methods for drug combinatorial discovery, but these methods are often tedious, expensive, and labor intensive [7]. In the past few decades, efficient approaches like microarray, next-generation sequencing, and multi-omics data have been developed to solve this problem [8,9]. As the number of potential drug components increases, the number of potential drug and dose combinations increases exponentially. Thus, systematically screening all possible drug combinations is not feasible [10]. Therefore, the search space for drug combinations requires appropriate computational methods urgently.

**Figure 1:**
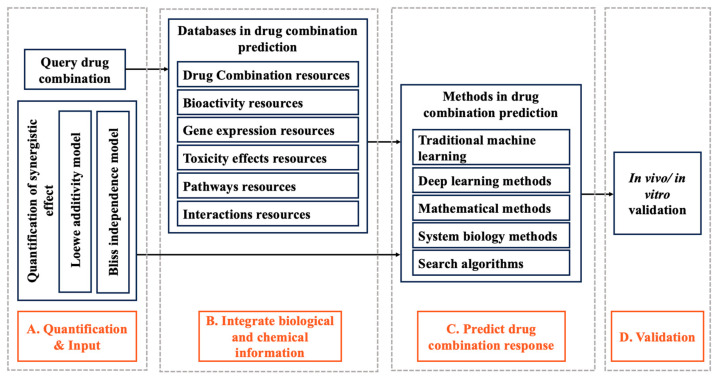
The workflow of the synergy scoring system of the drug combination. In predicting drug combination response, the model calculates synergistic scores according to the quantification of synergistic effect, input biological data and chemical information using a specific synergistic quantification method. Finally, in vitro/in vivo experiments were carried out.

As shown in Figure 1, the synergy scoring system is broken down into four sections according to the flow of the prediction model. Section A clearly explaining the concept of synergism and antagonism between drug combinations, then input the query combinations. Section B provides information on associated databases, such as gene expression and synergistic drug combination databases. Section C introduces several useful computational methods, including traditional machine learning, deep learning (DL), mathematical methods, systems biology methods and search algorithms. Finally, the experimental validation will be needed, illustrated in Section D.

## 2. Quantification of Synergistic Effect

**Figure 2:**
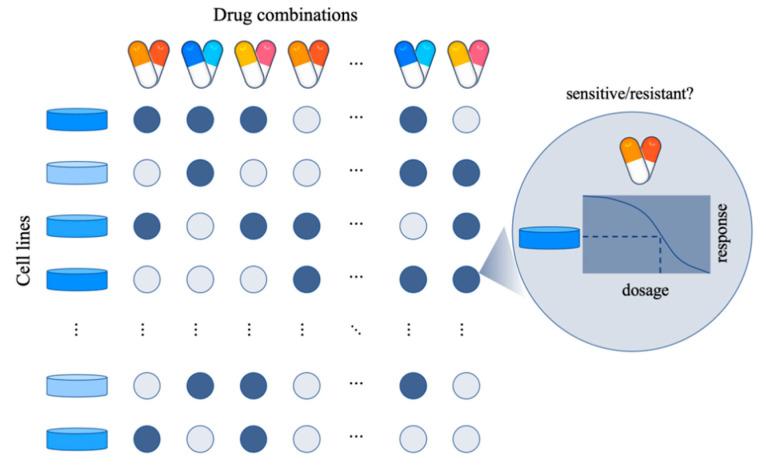
The overall framework of drug combination response prediction model. The principle of predicting drug combination response is to use the information provided by the database and advanced computational models to predict the synergy score of a specific cell line for a specific drug combination.

The problem of predicting drug combinations is usually defined as a classification or regression task. According to the definition, combined effects can be divided into synergistic, additive, and antagonistic effects because their effects are superior to, equal to, or inferior to the sum of the effects of each drug, respectively [11]. In the regression task, the basic assumptions for quantifying the synergistic or antagonistic effects of drug combinations are different according to the models [12]. The most used models in vivo and in vitro methods are the Loewe additivity model (Loewe) [13] and the Bliss independent model (Bliss) [14]. The overall structure of the drug combination response is shown in Figure 2.

### 2.1. Loewe Additivity Model

The Loewe additivity model defines the effect of a compound in combination with its combined effect. Synergistic and antagonistic effects are defined by Loewe as deviations from strict additive behavior. The doses of drugs 1 and 2 for the combination should be $d_{1}$ and $d_{2}$. $D_{1}$ and $D_{2}$ represent the dose of drugs 1 and 2 required to achieve the combination effect when used alone. Loewe [15] argues that where no interaction occurs between drug 1 and drug 2, the additive behavior of the drug combination takes the form of:

(1) $$\frac{d_{1}}{D_{1}}+\frac{d_{2}}{D_{2}}=1.$$

Similarly, when there are *N* non-interacting drugs, the Loewe additivity model for this combination is defined in this manner:

(2) $$\sum_{i=1}^{N}\frac{d_{i}}{D_{i}}=1,$$

where $d_{i}$ is the dosage of the i drug in the compound, and $D_{i}$ is the equivalent dosage of each drug that achieves the same effect when used alone.

When a combination of drugs has a synergistic effect, the same therapeutic effect can be achieved with a smaller dose than with a single drug. Therefore, the synergism is defined as:

(3) $$\sum_{i=1}^{N}\frac{d_{i}}{D_{i}}<1.$$

In contrast, in situations where a single drug is more effective than a combination of drugs, the antagonism is defined as:

(4) $$\sum_{i=1}^{N}\frac{d_{i}}{D_{i}}>1.$$

**Figure 3:**
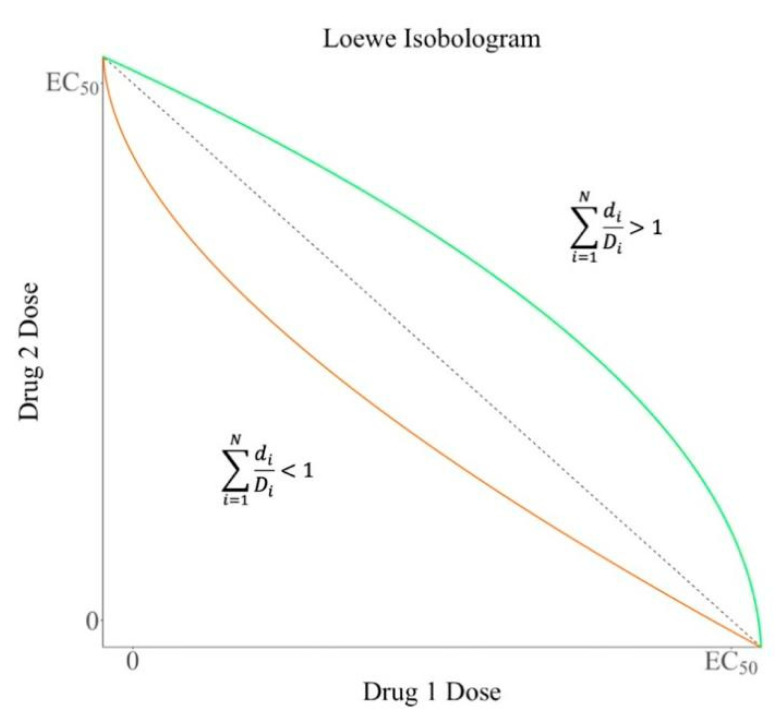
The isobolographic of Loewe additivity model. It shows the biological effects of drug combinations at different doses. The yellow curve shows the synergistic effect, and the green curve shows the antagonistic effect.

To better understand the Loewe additivity model, Figure 3 shows a graph in Cartesian coordinates that represents the dose-response relationship between the two drugs in a combination based on isolines. It portrays curves comprising many dose pairs that achieve a specific drug response effect. Each line represents a dose combination for a particular drug pair. It can clearly distinguish the effects of different drug combinations at different doses.

### 2.2. Bliss Independence Model

The Bliss independent model, a classic approach to quantifying drug combination effect, considers the two drugs in the combination are probabilistically independent when used alone. Therefore, using the independent definitions of probability and statistics, the consequences of a combination can be determined [16]. According to Bliss, the reaction of each component’s concentration can be used to predict the response. E is the effect of the combination, while $E_{1}$ and $E_{2}$ are the fractional effects (between 0 and 1) produced by consuming drug 1 and drug 2 separately. Thus, the effect that drugs 1 and 2 have when combined can be formulated as:

(5) $$E=E_{1}+E_{2}-E_{1}E_{2}.$$

A synergistic effect occurs when E is greater than the right side of the equation. Otherwise, there is animosity when E is less than the right side of the equation.

These two fundamental various strategies, Bliss and Loewe, have been commonly utilized for co-exposure tests. Nonetheless, at times, they might introduce decisively various outcomes that relate to the dose-response of a solitary medication. There are many investigations contrasting the Bliss independence model with the Loewe additivity model [17,18,19]. In these comparisons, the overall biological plausibility of the Loewe additivity model makes it slightly preferable. Specifically, when two drugs interact with the same pathway or target, Loewe expects the combined action to be better, while the Bliss independence model aims for non-interacting drug combinations. It is still unclear which model is suitable for studying the combined effects of drugs, and model selection is a major issue. Bliss may misjudge synergism, while Loewe may overemphasize antagonistic effects. One of the most significant obstacles in the field remains the lack of consensus among researchers regarding the precise quantification and definition of synergies and antagonistic relationships [20].

## 3. Databases in Drug Combination Prediction

Numerous omics databases have been created in response to the growth of systems biology and molecular biology. Table 1 lists some important databases of five types pertinent to recognizing effective drug combinations, including synergistic drug combination, bioactivity resources, gene expression, toxicity/off-target effects, pathways resources and interactions resources. These omics data are typically tested on various cell lines, using large numbers of single drugs or drug combinations, and have received robust experimental validation. Using these omics data, researchers can develop more efficient computational models of drug combinations to accelerate the development of clinical therapies.

### 3.1. Drug Combination Resources

Drug Combination resources mainly include DrugComb, DrugCombDB, SYNERGxDB, NCI-ALMANAC, etc., to collect information about anti-cancer drug combinations. There is a significant overlap between the first three databases because some of their data derives from NCI-ALMANAC.

DrugComb [21] is a community-driven data portal for storing and analyzing drug combination and monotherapy screening data. It offers network modeling tools to picture the system of activity of a drug or combination of drugs for a specific disease sample. DrugComb database contains 8397 unique drugs, 2320 cell lines representing 33 tissues and over 750 thousand unique drug combinations obtained from 37 studies. In addition, DrugComb also provides these combinations with five different types of synergy scores, including Bliss, HSA, Loewe, ZIP and S scores. In MatchMaker, a model proposed by Kuru, H.I. et al. [41] synergy scoring data provided by the DrugComb is used. The model has three layers of network architecture, two layers of drug-specific subnetworks (DSNs) and one layer of synergy prediction network (SPN).

DrugCombDB [22] is another web-based drug combination that integrates multiple data sources and drug combinations, including high-throughput screening analysis of drug combinations, external databases, and manual management of PubMed literature. This database contains more than 6.8 million experimental data with quantitative dose-response and concentrations of drug combinations encompassing 2 thousand drugs and 124 human malignant growth lines.

NCI-ALMANAC [23] database is an enormous matrix of combinations of antineoplastic agents. It has tested over five thousand combinations of 104 approved drugs and measured synergies against 60 cancer cell lines, resulting in more than 290 thousand synergies scores. The study by Sidorov P. et al. [42] was modeled on a dataset provided by NCI-ALMANAC to predict synergy scores for each NCI-60 cell line. They used the Random Forest (RF) algorithm and the Limit Gradient Lift (XGBoost) algorithm to build 2 separate models for each cell line.

SYNERGxDB [24] is a cloud-based pharmacogenomics portal that identifies synergies by incorporating numerous high-throughput drug combination studies with sub-atomic and pharmacological profiles of an enormous board of malignant growth cell lines. Additionally, it provides analytical tools for predicting biomarkers across cancers and identifying successful treatment combinations.

### 3.2. Bioactivity Resources

ChEMBL [25] is a chemical database of bioactive molecules with drug-like properties that have been manually curated. The current version of the CHEMBL database contains more than 2.3 million distinct compounds, 15 thousand protein targets and 20 million bioactivity measurements. Ye, Z et al. [43] proposed ScaffComb, a deep learning framework that can be applied to ChEMBL databases for virtual screening of drug combinations in enormous synthetic information bases.

DrugBank [26] is a web-enabled database that combines specific information about drug information with thorough drug target information. DrugBank included data on 2358 small molecule and biotechnology drugs, 4563 drug targets, 497 drug metabolizing enzymes and drug transporters, and 2242 compound drug-target binding constants. Ke, J. et al. [44] searched for candidate compounds and aspirin target information from DrugBank to find drug combinations with antiplatelet effects. They finally verified the synergistic effects of Ginkgo biloba extract.

PubChem [27] contains more than 115 million unique chemical structures, 306 million chemical entities, 304 million biological activity data points and 204 thousand interactions between chemicals, genes, and proteins. Unlike DrugBank, which has detailed drug information, PubChem is more like ChEMBL, which focuses more on chemical information.

### 3.3. Gene Expression Resources

In drug synergistic studies, Gene Expression Omnibus (GEO) [28] can query gene expression, including expression chip data, genome methylation, genome-protein interaction, etc. Lv, Y. et al. [45] used the GEO database to collect relevant gene expression and clinical data for osteosarcoma and para-cancerous tissues while investigating drug response prediction for osteosarcoma.

A database named the Library of Integrated Network-Based Cellular Signatures (LINCS) allows for comparisons of cell expression profiles or other cell processes before and after cell perturbation by various methods, mainly including CMap-based L1000, Drug Toxicity Signature Generation Center (DToxS), etc. Aissa, A.F. et al. [46] used an established preclinical model of non-small-cell lung carcinoma (NSCLC) to analyze recognized markers utilizing LINCS to foresee and validate the function of small molecules.

Connectivity Map (CMap) [29] is a database created by the LINCS Center for Transcriptomics at the Broad Institute using the L1000 sequencing method, primarily used to demonstrate the functional connection between genes, disease states, and small molecule compounds. To save the sequencing cost, only 978 representative landmark genes were sequenced during the sequencing process, and the expression level of the remaining 11,350 genes was predicted by advanced algorithms. Jin L. et al. [47] proposed a CMAP-based scoring framework for predicting new adaptation diseases for drug combinations. In this framework, CMap gives an information-driven way to deal with the recognition of the relationship between genes, diseases, and drugs.

### 3.4. Toxicity Effects Resources

Side Effect Resource (SIDER) [30] database that integrates information on drugs, targets, and drug side effects. It provides a platform for users to understand the effects of drugs and their adverse reactions fully. It also provides relevant information about the indications for the drug. Prinz, J. et al. [48] proposed a novel machine-learning approach that combines data from SIDER and GWASdb databases into a joint matrix. The model could be used to develop treatments with fewer side effects and test new indications for existing drugs.

TOXRIC [31] provides information on toxicological/feature data, Machine Learning (ML)-ready sub-datasets visualization of multiple benchmarks, etc. More than 113 thousand compounds, 13 toxicity datasets and 39 feature types are included in the TOXRIC data.

Tox21BodyMap [32] is an intuitive web tool that supports rapid chemical toxicity assessment and mechanism hypothesis generation. It gives a perception of mapping Tox21/ToxCast assay targets to the districts of the human body. The web server visually displays chemobiological activity patterns by mapping assay targets to organ systems.

### 3.5. Pathways Resources

KEGG Pathways [35] is a compilation of human responses and biological pathways, which can be used in drug combination prediction. Like KEGG, Reactome [33] is a database of peer-reviewed articles written by experts on responses and biological pathways in the human body. Compared to KEGG, it is an improved search and data mining tool that simplifies the data search and study related to biological pathways. The library currently covers pathways that concentrate on 19 species, including classical metabolic pathways, signal transduction, gene transcription regulation, and disease. In addition, it uses more than one hundred distinct online bioinformatics resources, such as the NCBI, Ensembl, and UniPro. To reveal the synergistic mechanism of natural products and anti-tumor drugs in the therapy of cancer, Chamberlin, S.R. et al. [49]. Considered pathways in the Reactome database targeted by natural products. They found a significant increase in coverage in the Reactome database relative to other databases, such as Cancer Targetome, that collected FDA-approved cancer drugs in the covered pathways. Moreover, as an interactive database, Pathbank [34] provides information on associated organelles, chemical structures, subcellular compartments, protein complex quaternary structures, and more.

### 3.6. Interactions Resources

The therapeutic target database (TTD) [36] contains a large amount of drug-related information on drug targets and natural product sources. Currently, TTD has a collection of more than 3 thousand targets and 30 thousand targeted binding drugs. Li, P. et al. [50] used the TTD database to develop a comprehensive model that can be used to study the mechanism of the compound Danshen formula (CDF).

Bingding DB [37] is a publicly accessible database that primarily collects affinity interactions between drug target proteins and small drug-like molecules. It is collected from US patents, scientific publications, and other databases such as PubChem, ChEMBL, etc. The Human Protein Reference Database (HPRD) [38] is the largest database of human protein interactions. The STRING database [39] incorporates all known and anticipated relationships between proteins. This database contains more than 14 thousand organisms, 67.6 million proteins, and 20 billion interactions.

Search Tool for Interacting Chemicals (STITCH) [40] can be used in predicting interactions between chemicals and genes. It is cross-linked with databases such as BindingDB. It shares protein data with STRING, a gene-association database developed by the same team. STITCH collects data from human annotation databases, including DurgBank, TTD, KEGG, Reactome, and ChEMBL. Wang T. et al. [51] used a variety of advanced computational methods to build effective predictive models. They extracted topological characteristics of each drug combination’s topology using a drug network built from STITCH.

## 4. Methods in Drug Combination Prediction

Over the course of the last many years, computational techniques have been broadly used to predict drug combinations, including traditional machine learning methods, deep learning methods, mathematical methods, systems biology methods, and search algorithms. A brief description of each method reviewed is listed in Table 2. Traditional machine learning applies to various feature types for high prediction accuracy in different scale databases. For a long time, traditional machine learning has been applied to improve and optimize drug discovery and design processes and integrate with other computational methods [52,53]. Deep learning methods can learn the complex nonlinear relationships between input attribute data (such as genomics) and the associated output (such as synergy score) [54]. Due to its multi-processing layer, the accuracy of deep learning models will be incredibly improved with the increment of input data, particularly huge databases [55]. The key step of the mathematical model is to collect the necessary kinetic parameters from the literature or experiments. When cellular pathways and parameterization are available, mathematical simulations can be highly accurate for combinatorial drug discovery [56]. Systems biology methods analyze the therapeutic effects of drug combinations through various biological networks, which take a lot of biological knowledge [57]. Search algorithms are seen as a method that endeavors to investigate feature spaces, using the high performance of computers to purposefully exhaust some or all possible scenarios of a problem-solving space [15].

### 4.1. Application of Traditional Machine Learning in Drug Combination Prediction

Traditional ML methods include Support vector machine (SVM), Decision tree (DT) and Gradient boosting (GB). SVM is often used for classification tasks, where the goal is to find hyperplanes that separate positive cases from negative cases. Like SVM, a Decision tree is a tree-shaped predictive model that judges the feasibility of various situations based on known probability of occurrence. Different from the previous two methods, Gradient boosting is an ensemble algorithm. It obtains a subset of the sample by operating the sample set and then generates a series of base classifiers. These algorithms are good classifiers for identifying whether drug combinations belong to synergistic or antagonistic effects.

Support vector machine [58] is a sparse and robust classifier. SVM is very well at identifying subtle patterns in complex data sets. SVM also introduces kernel functions for faster computation and prediction of nonlinear problems. In addition, because SVM classifies by maximizing the interval, it is robust to noise and outliers. As mentioned above, the predictive model constructed by Wang T. et al. [51] utilizes SVM to obtain the best characteristic. Their results show that the best SVM classifier they built is significantly better than one that uses only individual features, with prediction accuracy (ACC) of 0.903 and Matthew’s correlation coefficient (MCC) of 0.806. This makes sense because their classifier combines several topological information about the drug. The model is expected to be a helpful method for predicting new drug combinations that didn’t exist in the training set. On large-scale data sets, SVM training time is very slow and takes up a lot of computing resources. What’s worse, SVM is poorly interpretable because its decision boundaries are determined by the support vector rather than all training samples [68]. In general, SVM has relatively high accuracy, strong generalization ability when dealing with nonlinear data and can capture complex relationships in the data.

Decision tree [59] is easy to handle and implement. Random Forest is a Bagging integration algorithm composed of decision trees. The results of the decision tree can be visually displayed through the tree structure, which is easy to understand and visualize. Wu, L. et al. [69] built an advanced deep forest-based model, ForSyn. ForSyn is a multi-layer cascade structure with two new forest types embedded in each cascade as units. Comparing ForSyn with other advanced algorithms on several datasets, their results show that ForSyn performs better, with an area under the precision–recall curve (AUPR) of 0.591 and recall of 0.537. Unlike traditional machine learning methods, this model solves problems about the imbalance of data types and high dimensions of characteristics. However, the model is still confused by the scale of input data.

Moreover, its ability to generalize to new anti-cancer drugs or cancer cell lines is insufficient. The intrinsic problems with these drug combination predictions remain unresolved. Decision trees are prone to over-fitting training data, especially when the depth of the tree is large or there are too many leaf nodes [70]. The accuracy of decision trees mainly depends on data quality, feature selection, tree structure and parameters. In cases where there are complex interactions between complex datasets and features, the accuracy of the decision tree may decline.

Gradient boosting [60] improves the accuracy of any given learning algorithm. It shows high predictive accuracy in many machine learning tasks and is particularly good at dealing with nonlinear relationships and high-dimensional data. It can clarify the decision-making process for predicting the outcome by looking at the importance of each weak learner. Xu, Q. et al. [71] introduced a new model based on the stochastic gradient boosting algorithm called PDC-SGB. The model constructs 732-dimensional feature vectors containing biological, chemical, and pharmacological information for each drug combination. This study integrated six types of characteristics to describe drug combinations, including molecular two-dimensional structure, structural similarity, anatomical therapeutic similarity, protein-protein interactions, chemical-chemical interactions, and disease pathways. Compared with other advanced models, this model shows better performance and feature prediction ability, with its AUC up to 0.9775. However, the performance of the biological part of the model is relatively low, which may be due to the incomplete molecular network or biological pathway and the oversimplified characterization of biological features. Unfortunately, gradient-boosting algorithms are prone to overfitting on training sets, especially when there are many weak learners. The algorithm also involves more hyperparameters than other methods. As a result, hyperparameter tuning is more complex [72].

### 4.2. Application of Deep Learning Methods in Drug Combination Prediction

Deep learning models mainly contain Feedforward neural network (FNN), Autoencoder (AE), Graph neural network (GNN) and Deep belief network (DBN). In the Feedforward neural network, every neuron is organized in layers, and every neuron is simply associated with the neurons of the past layer. It is often used as a baseline for Deep learning methods. Unlike FNN, the autoencoder is more complex and is a semi-supervised and unsupervised learning artificial neural network for reduction and anomaly detection. The graph neural network uses deep learning to directly learn graph structure by extracting and discovering its characteristics. Deep belief networks can be utilized not exclusively to recognize characteristics and classify data but additionally to produce data.

A feedforward neural network [61] receives the outputs of the past layer and results it to the following layer without feedback. The advantage of this model is that it has strong nonlinear modeling ability and can automatically learn complex relationships between input features. In addition, it can also improve the performance of the model by increasing the number of hidden layers and neurons. Tsai, P.L. et al. [73] proposed a multi-layer FNN with two hidden layers, which was used to predict the treatment outcome of antidepressant therapy in patients with initial treatment and first diagnosis of major depressive disorder (MDD) patients during the severe depressive stage. The first layer of the neural network is the input layer, where each unit receives a one-dimensional data vector containing patient characteristics. The final layer is the output layer that performs the classification. The evaluation results show that the model has an Area Under Curve (AUC) range from 0.7 to 0.8 and can use clinical features and peripheral biochemical characteristics to predict the outcome of antidepressant therapy. The drawback is that during the model training process, they used a small sample size and could not carry out a more detailed analysis. Deep neural networks still have insufficient mechanisms to explain the interactions between variables. Besides, it requires many training samples and a complex network structure, which is easy to overfit.

Furthermore, the training process of feedforward neural networks takes a long time, especially when dealing with large data sets [74]. The results of feedforward neural networks often lack interpretability. The accuracy of this method is affected by many factors, including data quality, feature selection, network structure and hyperparameter selection.

Autoencoder [62] includes both encoder and decoder, a representation learning algorithm in a general sense. It has a strong feature learning ability and can extract useful features from drug response data through unsupervised learning without the need for manually labeled information [75]. Liu, Q. et al. [76] constructed a knowledge-enabled and self-attention transformer-boosted deep learning model, TranSynergy. It includes three major components: (1) input dimension reduction component, (2) self-attention transformer component, and (3) output fully connected component. Their experimental results of model evaluations showed that TranSynergy outperformed the most advanced approaches, and the AUC and AUPR reached 0.908 and 0.625, respectively. As with traditional computational models, the TranSynergy model selected only a few cancer-related genes that included drug targets and annotations due to limited training data. In addition, the model will also cause dimensional disasters due to too many feature dimensions, resulting in overfitting problems. Autoencoder have the risk of overfitting when dealing with large-scale drug response data, especially when the training set is small. The training process of the autoencoder model is unsupervised, so the features extracted by the model are often difficult to interpret [75,77].

Graph neural network [63] is an emergent framework that has emerged recently. The advantage of a graph neural network is that it can capture the relationship and topological information between the nodes in the graph and transform the data into low-dimensional and more discriminative feature space. In addition, graph neural networks can automatically learn the feature representation of nodes and edges [78]. Wang J. et al. [79] proposed a graphical neural network (GNN) and attention mechanism-based model called DeepDDS. In this model, the chemical structure of the drug is represented by a graph. The drug embeddings are calculated according to the above two deep learning models. By integrating genomic and drug signatures, DeepDDS can capture important information from drug chemical structures and gene expression patterns to identify synergistic drug combinations that target specific cancer cell lines. Additionally, they compared DeepDDS with deep learning methods and traditional machine learning methods on a benchmark dataset. Finally, the results demonstrate the better performance of DeepDDS compared to other models, and its performance measures of AUC, area under the AUPR and accuracy reach 0.93, 0.93 and 0.85, respectively. Similarly, DeepDDS still did not show satisfactory predictive accuracy on independent test sets for the same reason described earlier. The main disadvantages of graph neural networks are as follows: (1) Due to the complex structure of GNN, its model training process is relatively difficult. (2) GNN is also a black box, which makes it difficult to explain its decision-making process. (3) GNN is vulnerable to adversarial attacks, and its robustness needs to be improved [80].

A deep belief network [64] can train the weights between its neurons, allowing the whole network to generate enough training data to maximize the probability. Moreover, DBN can automatically learn high-level abstract features from data through unsupervised learning and perform back-propagation through supervised learning. When it comes to supervised training with just some labeled data and extracting features from regular data, DBN performs admirably [81]. Chen, G. et al. [82] introduced a stacked restricted Boltzmann machine (RBM), which can predict the response of drug combinations from gene expression, pathways, and body fingerprints. In their model, the training data is utilized before the learning stage to optimize the weight of the input using contrastive divergence. Their evaluation of the model showed an accuracy rate of 71.5%, the recall of 60.2%, and an F score of 65.4%. Overall, they performed better than the DREAM competition group. The RBM model also faces the problem of data integrity and lack of experimental data, which may be the cause of model performance degradation. Moreover, DBN is prone to over-fitting when dealing with small sample data, so some regularization methods are needed to alleviate over-fitting [81]. DBN can achieve high accuracy in drug response prediction models, but the training data and hyperparameter selection need to be carefully considered in practical applications.

### 4.3. Application of Mathematical Methods in Drug Combination Prediction

Mathematical methods include Network analysis and Dynamic mathematical models. Network medicine uses a systems-network perspective to understand the disease mechanism [65]. Similarly, the Dynamic mathematical model studies the effects of drug combinations on potential protein concentrations and drug combination therapies, which can effectively control the progression of disease states [15].

The human body is composed of a rich variety of biological units, and with the advancement of bio-measurement technology, various types of disease networks have been established. With the help of Network analysis, the complex interactions between drugs and proteins can be captured, including physical interactions, metabolic pathways, signal transduction, etc. In addition, it can provide an interpretation of the predicted results, for example, by analyzing critical paths in the network, node importance, etc. [83,84]. Yin N. et al. [85] explained the connection between network topology and the effect of drug combinations by displaying the interaction of drug combinations and their targets in the network. They found that the effect of drug combinations depends heavily on the network topology, and they were able to identify motifs that could serve as useful catalogs for rational drug combination design of enzyme systems. Unlike most studies on drug synergies, they focused on antagonism and synergies. Their model generally provides a rational and easy-to-apply approach to designing synergistic drug combinations. However, the results of this model are only based on enzyme networks, and other types of biological networks still need to be further explored. Moreover, this method requires a large amount of drug and protein interaction data, so acquisition and collation are challenging [83,84]. The accuracy of network analysis is like many other methods and is also affected by several factors, including data quality, network construction methods, and prediction algorithms.

The dynamic mathematical model captures important dynamic aspects of disease treatment. It can describe the processes of drug absorption, distribution, metabolism, and excretion in organisms, to simulate drug reactions more accurately. It also has a strong predictive ability, which can predict the effect of drugs under different doses and dosing schemes and support the individualization of drug therapy [86,87]. Geva-Zatorsky, N. et al. [88] dealt with accurately tracking different protein concentrations, considering different drugs through a dynamic proteomics method. They tracked down that the dynamics of proteins’ reaction to drug pairs can be accurately depicted through their responses to various drugs. However, Dynamic mathematical models are usually constructed based on a series of mathematical equations, and their complexity may limit the interpretability of the models [86,87]. These models typically have high accuracy but are more data-intensive, requiring more data and optimization of the model parameters.

### 4.4. Application of Search Algorithms in Drug Combination Prediction

The breadth-first search algorithm is always extended outward through the boundary between found and unfound vertices. The applications of breadth-first search, especially in drug structures, include finding the shortest path and the minimum distance between two points.

As one of the least complex graph search algorithms, the Breadth-first search algorithm is the basis of numerous graph algorithms. It can consider a large amount of potential drug-target interactions. Ji, L.S. et al. [66] investigated the immunomodulatory mechanisms of Bushen formula (BSF) combined with entecavir (ETV) in patients with newly treated chronic hepatitis B (CHB) and CHB patients with partial virological response to ETV. They finally demonstrated that the combination of these two drugs helped ETV partially alleviate hBsAg reduction in patients and is a potential treatment for these patients. However, in their study, the underlying immunomodulatory mechanisms underlying BSF treatment of CHB patients remain to be explored. This method is not robust enough. Moreover, because its prediction results are often based on the similarity between the drug and the target, it does not consider other factors such as drug metabolism, drug delivery, etc. [89,90]. Regarding the accuracy of the model, the feature selection, parameter selection and tuning, evaluation and verification methods of the search algorithm have a decisive influence.

### 4.5. Application of Systems Biology Methods in Drug Combination Prediction

Systems biology methods hypothesize that drugs that are effective for specific diseases can be used as candidates for other diseases with similar characteristics of changes in gene expression. The model is suitable for rapid drug combination prediction for experimental verification.

The cMap database provides gene expression profiles of numerous small molecules against different cancer cell lines, which provides rich data for the Signature-based model. In addition, Systems biology methods can integrate large-scale biological data at different levels, such as gene expression, protein interactions, metabolic pathways, etc., to gain a more comprehensive understanding of drug action mechanisms and influencing factors [91]. Kim J. et al. [67] have developed a web-based program called K-Map. The program can uncover the perplexing communications between protein kinases and their inhibitors and provide the basis for rational clinical drug use. This model can link kinases to drugs using quantitative signatures of kinase inhibitor activity. In addition, it is highly real-time and useful, and they also update their data on a quarterly basis, making it even more valuable. However, there are still some disadvantages to this method: accuracy is highly dependent on the quality of the input data. Moreover, the method usually uses complex network models and algorithms, which makes it difficult to interpret the result [91]. The systems biology approaches construct complex network models by integrating multiple data sources to achieve high accuracy.

## 5. Discussion

In clinical therapy, personalized cancer treatment requires that models predicting drug response can effectively predict the effect of the drug combination and provide reasonable explanations in the face of complex molecular characteristics and noisy pharmacogenomic data. Till now thousands of bioactivity databases and various computational methods have been generated in recent years. This review focuses on the databases and methods used to predict the response of cell lines to drug combinations, as well as the definition of synergies, to provide cancer patients with individualized, precise treatment regimens, which may improve patient survival and survival time and achieve precision cancer therapy.

Although many studies have already achieved great predictive performance, there are still many challenges in this research direction. Appropriate databases seem to be crucial for better predictive performance. For instance, in some large publicly available databases, the size of cancer cell lines and drugs is insufficient to train models with strong generalization. Moreover, most of these models make predicting the response of novel drugs or novel cell lines difficult, which didn’t appear in the training set. As mentioned in this article, the problem of class imbalance also needs to be solved, which is also an important reason for the low generalization ability of the model. In most of these models mentioned in this paper, structural information, physical and chemical information about drugs, and cell lines’ expression information are used as characteristics to predict the effect of drug combinations. The study of drug synergies should pay more attention to the biological links between drug combinations and cell lines rather than the characteristics of each. Therefore, more features of omics should be considered in the prediction process. Another issue worth exploring is whether the drug combination is synergistic or antagonistic, which is generally related to the drug dose. This can be simply understood as the fact that drug combinations are often found to be synergistic in one dose range and antagonistic in another. However, considering the different doses of drugs based on studying different effects of drug combinations is a very difficult problem. Although some studies [92] have considered the effect of drug dosage, the issue still needs to be explored more deeply to achieve the goal of precision medicine. In recent years, deep-learning language models have shown great promise in drug discovery, including understanding drug-drug interactions, protein design, and engineering. For example, ProGen, a language model produced by Madani A. et al. [93], can generate protein sequences with predictable functions and can be adapted to different protein families. Another example is the application of ChatGPT in predicting and interpreting common drug-drug interactions (DDIs) [94]. With the help of ChatGPT, clinicians and patients can effectively identify potential DDI effects and make the right decisions.

## Figures and Tables

**Table 1:** Important omics databases related to the drug combination.

Data Type	Database	URL	Latest Update	Description
Synergistic Drug Combination	DrugComb [21]	https://drugcomb.fimm.fi/ (accessed on 13 July 2023)	2021	Synergistic Drug Combination mainly contains data on the response of cancer cell lines to or combinations of drugs.
	DrugCombDB [22]	http://drugcombdb.denglab.org/ (accessed on 13 July 2023)	2019	
	NCI-ALMANAC [23]	https://dtp.cancer.gov/ncialmanac (accessed on 13 July 2023)	2017	
	SYNERGxDB [24]	https://www.synergxdb.ca/ (accessed on 13 July 2023)	2019	
Bioactivity resources	ChEMBL [25]	https://www.ebi.ac.uk/chembl/ (accessed on 13 July 2023)	2023	Bioactivity resources mainly contain the biological activity data of small molecules, drug targets, enzymes and proteins.
	DrugBank [26]	https://www.drugbank.com (accessed on 14 July 2023)	2023	
	PubChem [27]	https://pubchem.ncbi.nlm.nih.gov (accessed on 14 July 2023)	2023	
Gene expression	GEO [28]	https://www.ncbi.nlm.nih.gov/geo/ (accessed on 14 July 2023)	2013	It mainly includes gene expression data with or without perturbation, gene methylation data and some interaction data.
	CMap [29]	https://clue.io (accessed on 14 July 2023)	2021	
	LINCS	https://lincsproject.org/ (accessed on 14 July 2023)	2022	
Toxicity effects resources	SIDER [30]	http://sideeffects.embl.de/ (accessed on 15 July 2023)	2015	Toxicity effects resources mainly contain information about drugs and targets, as well as information on side effects.
	TOXRIC [31]	https://toxric.bioinforai.tech/ (accessed on 15 July 2023)	2022	
	Tox21BodyMap [32]	https://sandbox.ntp.niehs.nih.gov/bodymap/ (accessed on 15 July 2023)	2020	
Pathways resources	Reactome [33]	https://reactome.org/ (accessed on 15 July 2023)	2023	Pathways resources mainly contain various biological pathways that facilitate drug combination prediction.
	Pathbank [34]	https://pathbank.org/ (accessed on 15 July 2023)	2020	
	KEGG Pathways [35]	https://www.kegg.jp/kegg/pathway.html (accessed on 15 July 2023)	2023	
Interactions resources	TTD [36]	https://db.idrblab.net/ttd/ (accessed on 16 July 2023)	2023	It mainly contains information about drug targets and targeted drugs that interact with them.
	Bingding DB [37]	http://www.bindingdb.org (accessed on 16 July 2023)	2023	
	HPRD [38]	http://www.hprd.org/ (accessed on 16 July 2023)	2008	
	STRING [39]	https://string-db.org/ (accessed on 16 July 2023)	2021	
	STITCH [40]	http://stitch.embl.de/ (accessed on 16 July 2023)	2016	

**Table 2:** The descriptions of some computational methods in drug combination prediction.

Methods	Algorithms	URL	Characteristics	Reference
Traditional machine learning	Support vector machine	—	Do well in identifying subtle patterns in complex data sets; poor interpretability; run slowly on large data sets.	[58]
	Decision tree	https://github.com/Lianlian-Wu/ForSyn (accessed on 18 July 2023)	Display visually; easy to over fit; accuracy may decrease when processing data with complex relationships.	[59]
	Gradient boosting	—	Do well in handling nonlinear relationships and high dimensional data; easy to over fit; hyperparameters tuning is complex.	[60]
Deep learning methods	Feedforward neural network	—	Do well in handling nonlinear relationships and high dimensional data; easy to over fit; poor interpretability; processing large data takes a long time	[61]
	Autoencoder	https://github.com/qiaoliuhub/drug_combination (accessed on 19 July 2023)	Feature learning ability is strong; poor interpretability.	[62]
	Graph convolutional network	https://github.com/Sinwang404/DeepDDS/tree/master (accessed on 19 July 2023)	Being able to capture the relationship and topological information between the nodes in the graph, poor interpretability, and robustness is of concern.	[63]
	Deep belief network		Perform well in supervised study; easy to over fit; poor interpretability.	[64]
Mathematical methods	Network analysis	—	Be able to capture complex interactions; good interpretability.	[65]
	Dynamic mathematical model	—	Be able to simulate drug reaction more accurately; poor interpretability.	[15]
Search algorithms	Breadth first search algorithm	—	Be able to consider a large amount of potential drug-target interactions; robustness is of concern.	[66]
Systems biology methods	Signature-based model	https://tanlab.ucdenver.edu/kMap(accessed on 20 July 2023)	Understand drug action mechanisms and influencing factors more comprehensively; high requirements on data quality.	[67]

## Data Availability

This review did not generate new data. The information on databases and methods mentioned in this review can be found in the URL columns in Table 1 and Table 2, respectively.
